# Healthcare Infections Associated with Care and Treatment of Humans and Animals 

**DOI:** 10.3201/eid1412.081207

**Published:** 2008-12

**Authors:** James J. Gibson, Marion A. Kainer, Sarah E. Raskin, David J. Weber, Walter A. Orenstein, James M. Hughes

**Affiliations:** South Carolina Department of Health and Environmental Control, Columbia, South Carolina, USA (J.J. Gibson); Tennessee Department of Health, Nashville, Tennessee, USA (M.A. Kainer); University of Arizona, Tucson, Arizona, USA (S.E. Raskin); University of North Carolina, Chapel Hill, North Carolina, USA (D.J. Weber); Southeastern Center for Emerging Biologic Threats, Atlanta, Georgia, USA (W.A. Orenstein, J.M. Hughes); Emory University School of Medicine, Atlanta (W.A. Orenstein, J.M. Hughes)

**Keywords:** Healthcare infections, hospital infection, zoonosis, conference summary

On May 14–15, 2008, the Southeastern Center for Emerging Biologic Threats convened a conference in Atlanta, Georgia, of more than 60 public health officials, clinicians, and researchers from its 7 member states to discuss infections associated with the care and treatment of humans and animals (*1*). Participants discussed hospital infection reporting laws, research on the epidemiology and prevention of healthcare-associated infections (HAIs), and current infection control practices. Conference presentations are available online at www.secebt.org.

HAIs refer to the 1.7 million infections that are acquired, introduced, or propagated by personnel, visitors, and patients in human healthcare facilities. Key prevention methods include mandatory vaccination of personnel (unless a medical contraindication exists), consistent and appropriate infection control practices such as appropriate hand hygiene and judicious use of antimicrobial drugs, prompt detection of and responses to potential outbreaks, and routine surveillance. Surveillance is essential to appropriately focus resources and to evaluate effectiveness of prevention measures. More than half of the 50 states have passed mandatory reporting laws, many of which are unfunded mandates that threaten to overburden infection control professionals. These professionals are essential to conduct surveillance and to assist in the implementation and evaluation of prevention measures.

Public health and hospital professionals from 6 states described their experiences with mandatory hospital reporting; all collect data by using the National Healthcare Safety Network (NHSN). Of concern is the public’s emphasis on measuring HAIs versus measuring implementation of recommended infection control guidelines, given the variation of risks from institution to institution and the limitations of risk-adjustment in making reported infection rates comparable. Furthermore, postdischarge surveillance to determine infections with onset after hospitalization remains challenging. The experiences of these states demonstrate the importance of interacting with key stakeholders and legislators before enactment of the law. In addition to the use of process measures (e.g., Institute for Health Improvement “bundles,” which are evidence-based combinations of processes for infection prevention) to reduce ventilator-associated pneumonia and central catheter–related bloodstream infections, the group recommended securing dedicated time and resources for piloting data collection before statewide implementation and producing reports that are fair to hospitals and useful to consumers, third-party payers, and hospital personnel.

Compliance with data collection and reporting requirements requires substantial institutional resources. Validation studies performed by South Carolina and New York have demonstrated that, overall, hospitals were not underreporting infections during the initial inpatient stay or required readmission. There were, however, inconsistencies between medical records and NHSN data entry. Inconsistencies were particularly prominent in the following data elements: American Society for Anesthesiologists score, duration of procedure, endoscope use, and extent of surgical site infection (superficial, deep, organ-space). New York found that the following variables not taken into account for risk-adjustment by NSHN were associated with infection: body mass index, diabetes, female gender, immunodeficiency, and emergency procedure. Thirty-two percent of surgical site infections (SSIs) in patients who underwent coronary artery bypass graft surgery were detected during initial admission, 63% were detected on readmission, and 5% were detected by postdischarge surveillance. In contrast, 63% of colon surgery SSIs were detected during the initial admission, 24% upon readmission, and 13% on postdischarge surveillance (*2*).

Several legal-ethical issues regarding mandatory surveillance and the value-based purchasing process of Centers for Medicare and Medicaid Services (CMS) are of great concern. These issues include the potential for public misinterpretation of data; incentivizing “gaming” of the system (e.g., underreporting of infections, possible reluctance of clinicians to perform high-risk procedures); diverting resources from prevention and care to reporting; inappropriate use of antimicrobial drugs for patients for whom they are not recommended; and using benchmarks (e.g., administrative claims data) that have not been validated. Nevertheless, the ethical concern of preventing patient and personnel harm remains primary.

A strong business case can be made for reducing HAIs, which are costly because of increased lengths of hospital stay and associated opportunity costs (*3*). Measures of cost-effectiveness demonstrate that, at $2,000–$8,000/quality-adjusted life-year, infection control methods are cost-effective relative to other preventive health interventions (*4*). In addition, reported HAIs may become a factor in healthcare consumer decision-making and, therefore, practitioner revenues. The business case for improving HAI prevention measures is strengthened by 2 factors. First, beginning in October 2008, CMS will refuse to pay for 6 conditions that qualify as infections not present on admission. Second, recent research demonstrates that the preventable fraction of HAIs may be much larger than previously thought.

Emerging research suggests that implementation of effective infection control practices is crucial for reducing HAIs and is a topic that warrants further study. Multisite studies such as the Michigan Keystone Collaborative suggest that local adaptation of implementation methods, environments characterized by strong teamwork among clinicians, administrators, and care providers, and emphasis on best practices such as removing unnecessary lines are factors that can reduce HAIs (*5*,*6*). These studies also demonstrate the extent of HAI preventability. Implementation research requires a multidisciplinary approach including open-mindedness about social/behavioral science and methods, e.g., using complexity theory instead of randomized controlled trials (*7*). Collaborative approaches should be expanded beyond intensive care units and prevention of infection. There needs to be increased focus on prevention of transmission (e.g., *Clostridium difficile*, multidrug-resistant organisms).

Although research on HAIs in human healthcare settings requires innovation, research on HAIs in veterinary settings demands even more effort. Veterinary HAI research lacks consistent definitions, prevalence data, infection control guidelines, and measures of practice and other core elements of surveillance. Conducting research on veterinary HAIs is increasingly important, given the documentation in North America of pathogens of serious concern to humans and animals, including multidrug-resistant *Salmonella* spp., various herpesviruses and influenza viruses, *C*. *difficile*, and methicillin-resistant *Staphylococcus aureus*, including the USA300 strain.

Research is needed in several areas, such as variation in critical care unit infection rates, preventable fraction of HAIs, and infection control (e.g., hand hygiene). We need better methods for validating results; better measures for process, implementation, and risk adjustment; better methods to measure hand hygiene; and standardization of formats and codes for efficient electronic data exchange. In addition, studies of public reporting, including effects of reporting on quality of care, antimicrobial drug use and resistance, and patient outcome, will be valuable in responding to mandatory reporting legislation.
